# Coordination in strictly metric-free swarms: evidence for the existence of biological diversity

**DOI:** 10.1098/rsos.241569

**Published:** 2025-03-12

**Authors:** Y. N. Jia, Y. Guo, W. L. Zhang

**Affiliations:** ^1^ School of Automation and Electrical Engineering, University of Science and Technology Beijing, Beijing 100083, People’s Republic of China; ^2^ Key Laboratory of Knowledge Automation for Industrial Processes of Ministry of Education, Beijing, 100083, People's Republic of China; ^3^ Beijing Electro-mechanical Engineering Institute, Beijing 100074, People’s Republic of China; ^4^ UAV Technology Research Institute, Beijing 100074, People’s Republic of China; ^5^ Hiwing Aviation General Equipment Co. Ltd., Beijing 100074, People's Republic of China

**Keywords:** coordination consistency, strictly metric-free swarm, hierarchical mechanism, differential evolution algorithm, limited vision

## Abstract

Coordination serves as a crucial metric for analysing collective behaviour in complex systems. Given the prevalence of biological diversity, this study re-evaluated the coordination issue in strictly metric-free (SMF) swarms, incorporating both limited perceptual ranges and hierarchical dynamics. Initially, the study introduced a single-layer hierarchical SMF model that was optimized using differential evolution strategies. Our empirical findings suggest that the leader–follower set-up marginally enhances coordination uniformity, with larger groups requiring a subtler leadership gradient than smaller groups. In addition, a global perspective may not be necessary for effective swarming because a high level of coordination and consistency can be achieved regardless of the population size, as long as the visual angle is not less than $105^{\circ }$. Furthermore, we examine how varying leadership layers influence collective behaviour. The results demonstrate that smaller groups benefit from uniform directional strategies, whereas larger groups (over 600 individuals) favour stochastic leadership patterns. Notably, for all group sizes, multi-layered frameworks incorporating stochastic components surpassed traditional SMF models in terms of coordination efficiency. These observations reinforce the importance of biological diversity for the formation of natural groups.

## 1. Introduction

Collective behaviour, characterized by the aggregation of numerous entities, whether living or non-living, is widespread in nature, including bacterial clusters, bee swarms, bird flocks and fish schools. The intricate biological coordination capability creates captivating displays, and the basic principles of swarm intelligence are considered useful tools to guide the engineering tasks of complex systems, such as sensor networks [1], formation control [2], transportation dispatch [3] and swarm control [4].

Models play a crucial role in uncovering the behavioural mechanisms that drive collective coordination processes. Classic swarming models, such as the Boid model [5], Vicsek model [6] and the Cucker–Smale model [7], typically rely on metric-based interaction rules. However, Ballerini *et al*. [8] revealed that interactions among birds in three-dimensional flight are governed by topological relationships, not by metric rules, which significantly enhance flock cohesion in response to external disturbances [8]. Similarly, Cavagna *et al*. [9] constructed a comprehensive three-dimensional model of starling flocks, empirically demonstrating the metric-free nature of animal behaviour correlation [9].

Building on these findings, Pearce *et al*. [10] proposed a strictly metric-free (SMF) three-dimensional swarming model in which individuals adjust their behaviours solely based on information from their topological neighbours [10]. Furthermore, Lewis & Turner successfully mirrored the observed changes in the starling density using a topological structure model [11]. Recognizing the absence of a characteristic length in bird flock interactions, Lewis *et al*. [12] introduced and analysed two topological swarming models [12]. Rahmani *et al*. [13] explored the dynamics of topological swarming models in spatially heterogeneous environments [13]. Expanding on the SMF model, Li *et al*. [14] developed a three-dimensional restricted vision field metric-free model by incorporating a limited vision mechanism and optimal visual field angle [14]. Yan *et al*. [15] suggested that topological optimization in multi-agent systems facilitates the straightforward establishment of various rules governing agent swarming behaviour [15].

Studies have identified hierarchical mechanisms as pivotal in driving collective behaviour. A significant advancement occurred in 2002 when Couzin *et al*. [16] enhanced the Boid model by introducing a more realistic yet simple rule to simulate the three key patterns in collective dynamics [16]. This model was further expanded into a hierarchical framework incorporating leaders within a group that possessed critical information and guided collective movements [17]. Subsequent studies, such as those by James *et al*., have demonstrated that leader–follower dynamics significantly influence pigeon group behaviours [18]. Chen *et al*. [19] deepened this understanding by uncovering a dynamic leader-switching mechanism during pigeon homing flights using high-resolution GPS data analysis [19]. Jia *et al*. [20] further found that hierarchical models exhibit superior quantitative synergistic performance compared with egalitarian models [20]. Therefore, incorporating hierarchical mechanisms is essential for developing effective swarming models.

Visual information is another crucial factor for individuals in a cluster when making collective decisions [21,22]. Traditional flocking models, such as the Vicsek and SMF models, often assume a $360^{\circ }$ field of view for each individual within a group. However, this assumption contrasts with natural observations, in which individuals typically have a limited field of view. For instance, starlings have a visual angle of 143°, pigeons 158° and owls 100.5° [16,23,24]. Generally, research has focused on enhancing the efficiency of the Vicsek model by modifying interaction rules or incorporating limited vision. For example, Wang *et al*. [25] explored methods to reduce the time required to achieve directional consistency in the two-dimensional Vicsek model by considering neighbour weights and introducing limited vision [25]. Lu *et al*. [26] improved the rate of achieving a consistent state in the Vicsek model by considering only partial neighbour information and specifically focusing on neighbours with higher degree values [26]. Additionally, Zhang *et al*. [27] proposed a modified approach in which particles have limited vision and continuously shift lines of sight, which not only increases the collaborative velocity of particles in the Vicsek model but also enhances its robustness [27]. To develop a more realistic physical swarming model, this study aimed to introduce a novel SMF model that incorporates both a limited field of view and a hierarchical mechanism. This integration was designed to enhance the realism and effectiveness of the model in accurately replicating the intricate dynamics of natural biological groups.

As collective behaviour models increase in complexity, identifying optimal parameters becomes increasingly crucial. The ultimate objective function presents a significant challenge in the absence of a specific mathematical formula. To address this, evolutionary algorithms such as the genetic algorithm [28], evolution programming [29] and evolution strategy [30] have become essential owing to their efficiency and simplicity. Among these, differential evolution (DE), introduced by Storn & Price, stands out for its multi-objective optimization capabilities in multidimensional spaces [31]. DE has been effectively applied across various fields, including image processing [32], signal processing [33], computer networks [34], artificial neural networks [35] and multi-objective optimization [36], demonstrating its effectiveness as a heuristic search technique with advantages, such as rapid convergence, minimal control parameters and robust optimization capabilities [37].

This study explores the impact of hierarchical mechanisms and limited vision on the coordination efficiency of multi-agent systems governed by the SMF model. Key findings include an improvement in system consistency through the incorporation of leader-follower dynamics, with leaders positioned on the convex hull. Optimization using the DE algorithm indicates that smaller populations benefit from larger preference direction weights, whereas larger populations require smaller weights. The integration of limited vision into the hierarchical model identified an optimal visual angle of 105° across various population sizes. Additionally, multi-layer hierarchical SMF models demonstrate superior coordination consistency, particularly in populations exceeding 600 individuals, compared with classical and single-layer models. This study highlights the effectiveness of these mechanisms in enhancing coordination in complex systems and reinforces the pervasive influence of biological diversity in natural assemblages.

## 2. Material and methods

### 2.1. Singular-layer hierarchical strictly metric-free model

#### 2.1.1. Modelling and simulation

First, we introduce the singular-layer hierarchical SMF (SLH-SMF) model characterized by three key parameters: edge intensity $\phi_{e}$, noise intensity $\phi_{n}$ and the weight of the leader’s preference direction ω. Therefore, the SLH-SMF model is defined as follows:


(2.1) $$r_{i}^{t+1}=r_{i}^{t}+v_{0}{\hat{v}}_{i}^{t},$$

(2.2) $$\begin{array}{lcr} & v_{i}^{t+1}=\left(1-\phi_{n}\right){\hat{u}}_{i}^{t}+\phi_{n}{\hat{\eta }}_{i}^{t} & \end{array}$$

and


(2.3) $$u_{i}^{t}=\left\{ \begin{array}{c} \left(1-\phi_{e}\right)\frac{{\left\langle {\hat{v}}_{j}^{t}\right\rangle }_{j\in B_{i}}\left(1-\omega \right)+\omega g}{\left|{\left\langle {\hat{v}}_{j}^{t}\right\rangle }_{j\in B_{i}}\left(1-\omega \right)+\omega g\right|}+\phi_{e}{\left\langle {\hat{r}}_{ij}^{t}\right\rangle }_{j\in S_{i}\;}\;\;\;\;\;\;\;\;\;\;\;\;\;\;i\in C\\ \frac{{\left\langle {\hat{v}}_{j}^{t}\right\rangle }_{j\in B_{i}}}{\left|{\left\langle {\hat{v}}_{j}^{t}\right\rangle }_{j\in B_{i}}\right|}\;\;\;\;\;\;\;\;\;\;\;\;\;\;\;\;\;\;\;\;\;\;\;\;\;\;\;\;\;\;\;\;\;\;\;\;\;\;\;\;\;\;\;\;\;\;\;\;\;\;\;\;\;\;\;\;\;\;\;\;\;\;\;\;\;otherwise \end{array}\right.,$$

where $r_{i}^{t}$ and $v_{i}^{t}$ represent the position and velocity of the individual i at time t, respectively. Vector ${\hat{r}}_{ij}^{t}$ points from particle i to particle j, where i,j∈[1,N] and N denotes the number of individuals in a cluster. Set $B_{i}$ includes the topological neighbours of the individual i, updated continuously based on agent i’s current position. Individuals on the convex hull form the set C. For an individual i within C, the topological neighbours on the convex hull are represented by set $S_{i}$, calculated as $S_{i}=B_{i}\cap C$. At time t, the noise affecting the individual i is represented by the random unit vector ${\hat{\eta }}_{i}^{t}$. The speed of each individual was constant and denoted as $v_{0}$. The symbol ∧ represents vector normalization and the angle bracket <> denotes averaging. Individuals on the convex hull were designated as leader individuals with a uniform preferred direction g and uniform weight ω. Because the SLH-SMF model described in this article is three-dimensional, the parameters $r_{i}^{t}$, $v_{i}^{t}$, $\eta_{i}^{t}$ and g are three-dimensional vectors.

**Figure 1.**
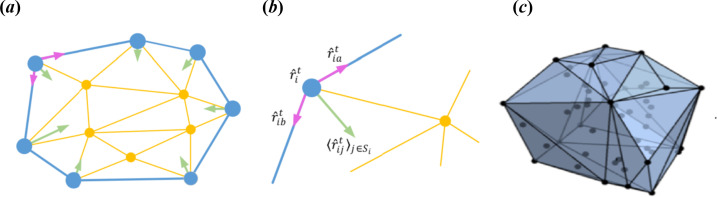
Topological structure diagram of the swarming model.

Figure 1*a,c* illustrates the neighbouring relationships among individuals, defined according to the topological structure using the Voronoi polygon. Figure 1*b* details the inward force ${\left\langle {\hat{r}}_{ij}^{t}\right\rangle }_{j\in S_{i}}$ on a marginal individual, which is the average of the unit vectors ${\hat{r}}_{ia}^{t}$ (pointing from the target individual to the adjacent individual a) and ${\hat{r}}_{ib}^{t}$ (pointing from the target individual to the adjacent individual b).

In the initial state of each experiment, N independent particles were randomly distributed within a cube of side length L. The velocity direction of each particle was also randomly generated, with all particles having the same velocity magnitude, $v_{0}$= 1. Thus, the initial position and velocity of particle i can be expressed as


(2.4) $$\begin{array}{lcr} & r_{i}^{0}=L\times [\zeta_{0},\zeta_{1},\zeta_{2}{]}^{T} & \end{array}$$

and


(2.5) $${\hat{v}}_{i}^{0}=[\zeta_{3},\zeta_{4},\zeta_{5}{]}^{T}-[0.5,0.5,0.5{]}^{T},$$

where i∈1,2,⋯,N and $\zeta_{k}(k=0,1,2,3,4,5)$ represents a random value in the interval (0,1).

The dynamic influence of the hierarchical mechanism within the SLH-SMF model was primarily observed through variations in the preference direction weight parameter ω. To assess the impact of ω on the coordination consistency of the collective system, we used the control-variable method. As detailed in our previous work [14,38], the edge intensity $\phi_{e}$ was fixed at 0.5 and the noise intensity parameter $\phi_{n}$ was set to 0.2 to simulate typical perturbations in real biological clusters. The degree of consistency achieved by the collective model was quantified using order parameter (or consistency parameter) P as a representative metric:


(2.6) $$P=|\frac{1}{N}\sum_{i=1}^{N}v_{i}|,$$

where the value of P is an indicator of the coordination state of the cluster. Each simulation was run for 1000 steps, but only the final 100 iterations were used to calculate P. To ensure the reliability of the experimental data, multiple independent repetitions of each experiment were conducted. The final consistency parameter value was the average *P*-value obtained from multiple independent trials.

#### 2.1.2. Parameter optimization

Given the numerous control parameters in the SLH-SMF model, optimal performance cannot be achieved by adjusting a single parameter alone. This study employs the DE algorithm to optimize the parameters of the SLH-SMF model, thereby enhancing the coordination consistency of the collective system.

The DE algorithm operates as follows: first, a new population is generated through mutation and crossover from the existing parent population. Subsequently, a greedy selection process is applied, in which each individual in the new population is compared with its counterpart in the parent population. Better-performing individuals are retained to form the final new generation.

##### 2.1.2.1. Initialization operation

An initial population of Np individuals was randomly generated. The current population $P_{t}$ can be expressed as


(2.7) $$P_{t}=\left\{x_{i,t}|x_{i,t}=\left(x_{i,t}^{1},x_{i,t}^{2},..x_{i,t}^{D}\right)\right\},i=1,2,...,Np,$$

where $x_{i,t}$ represents individual i at generation t, and each component $x_{i,t}^{j}(j\in \{1,...,D\})$ is initialized using a uniform random distribution in [0.1,0.9]. D represents the search space dimension. In the SLH-SMF model, there are three control parameters for optimizing $\phi_{n}$, $\phi_{e}$ and ω, so D=3. The population size Np is typically set in relation to D, often in the range [5D,10D]. For our model, the parent population size was set to 30, meaning Np=30, and $P_{t}$ consisted of 30 three-dimensional vectors. Each $x_{i,t}$ represents the *i*th vector containing a set of three control parameters.

##### 2.1.2.2. Mutation operation

The DE algorithm preserves the population diversity through mutations. In this process, individuals are randomly selected as parents with $x_{i,t}$ serving as the target vector in generation t. A classic differential mutation vector is given by


(2.8) $$V_{i,t+1}=x_{i1,t}+F\left(x_{i2,t}-x_{i3,t}\right),$$

where $V_{i,t+1}=\left[\upsilon_{i,t+1}^{1},\upsilon_{i,t+1}^{2},...,\upsilon_{i,t+1}^{D}\right]$, and i1,i2,i3∈{i=1,2,...,Np}(i1≠i2≠i3) denote three distinct individuals. The mutation factor F∈[0,1] scales the vector difference to control step size and prevent evolutionary stagnation. By randomly selecting these three individuals, this strategy ensures a balance between global and local exploitation in the search space.

##### 2.1.2.3. Crossover operation

Diversity is further maintained through a binomial crossover method, which combines each individual with its offspring variation vector $V_{i,t+1}$ to produce the trial vector $U_{i,t+1}=\left[u_{i,t+1}^{1},u_{i,t+1}^{2},...,u_{i,t+1}^{D}\right]$. Each component of the trial vector $U_{i,t+1}$ was selected with a certain probability from the offspring variation vector as described by equation (2.9):


(2.9) $$u_{i,t+1}^{j}=\left\{ \begin{array}{c} \upsilon_{i,t+1}^{j},\;\;\;\;\;\;\;\;\;\;\;{rand}_{j}\in [0,1]\leq CR\;\;or\;\;j=j_{rand}\;\\ x_{i,t}^{j},\;\;\;\;\;\;\;\;\;\;\;\;\;\;\;\;\;\;\;\;\;\;\;\;\;\;\;\;\;\;\;\;\;\;\;\;\;\;\;\;\;\;\;\;\;\;\;\;\;\;\;\;\;otherwise\; \end{array}\right..$$

where $\upsilon_{i,t+1}^{j}$ is the jth element of the mutation vector for the next generation; $x_{i,t}^{j}$ is the jth element of the target vector and ${rand}_{j}$ denotes a random number in [0,1]. The crossover probability factor CR determines the level of information exchange between the offspring, parents and intermediate mutants during the crossover process, typically ranging from 0 to 1. $j_{rand}$ is a random integer between 1 and D. Condition $j=j_{rand}$ ensures that at least one dimension of the target vector is inherited from the mutated vector.

##### 2.1.2.4. Selection operation

Optimization algorithms aim to minimize the fitness function value. Therefore, 1−P is chosen as the fitness function. The selection process employs a greedy algorithm that breaks down the optimization into several steps. At each step, the algorithm selects the individual with the smallest fitness function value from the current generation as the local optimal solution. This approach helps evolve and converge towards an optimal solution. During the selection operation, if the fitness function value of a newly generated individual is lower than that of the original individual, then the next generation individual is the original one. Otherwise, the original individual was retained. The next generation is determined as follows:


(2.10) $$x_{i,t+1}=\left\{ \begin{array}{c} U_{i,t+1}\;\;\;\;\;\;\;f\left(U_{i,t+1}\right)<\;f\left(x_{i,t}\;\;\right)\\ x_{i,t}\;\;\;\;\;\;\;\;\;\;\;\;\;\;\;\;\;\;\;\;\;\;\;\;\;otherwise \end{array}\right.\;\;\left(i=1,2,...,Np\right).$$

The process of mutation, crossover and selection continues until either the maximum number of optimization iterations is reached or the stopping criteria are met. At this stage, the algorithm outputs the optimal solution.

### 2.1.3. Model optimization

Most existing collective models, such as the Boid, Vicsek and previously proposed SLH-SMF models, assume that individuals have a field of view of 360°. However, this assumption does not reflect the reality of the natural biological groups. For instance, starlings have a field of view of 143°, and pigeons have a field of view of 158°. Consequently, these models do not accurately represent biological flocking phenomena because of the limited visibility of these birds. This discrepancy highlights the need to incorporate a limited vision mechanism into $360^{\circ }$ field of view flocking models.

To address these concerns, this study proposes an SLH-SMF model that incorporates a limited field of view as follows:


(2.11) $$r_{i}^{t+1}=r_{i}^{t}+v_{0}{\hat{v}}_{i}^{t},$$

(2.12) $$\begin{array}{lcr} & v_{i}^{t+1}=\left(1-\phi_{n}\right){\hat{u}}_{i}^{t}+\phi_{n}{\hat{\eta }}_{i}^{t} & \end{array}$$

and


(2.13) $$u_{i}^{t}=\left\{ \begin{array}{c} \left(1-\phi_{e}\right)\frac{{\left\langle {\hat{v}}_{j}^{t}\right\rangle }_{j\in B_{iv}}\left(1-\omega \right)+\omega g}{\left|{\left\langle {\hat{v}}_{j}^{t}\right\rangle }_{j\in B_{iv}}\left(1-\omega \right)+\omega g\right|}+\phi_{e}{\left\langle {\hat{r}}_{ij}^{t}\right\rangle }_{j\in S_{iv}\;},\;\;\;\;\;\;\;\;\;\;\;\;\;\;i\in C\\ \frac{{\left\langle {\hat{v}}_{j}^{t}\right\rangle }_{j\in B_{iv}}}{\left|{\left\langle {\hat{v}}_{j}^{t}\right\rangle }_{j\in B_{iv}}\right|},\;\;\;\;\;\;\;\;\;\;\;\;\;\;\;\;\;\;\;\;\;\;\;\;\;\;\;\;\;\;\;\;\;\;\;\;\;\;\;\;\;\;\;\;\;\;\;\;\;\;\;\;\;\;\;\;\;\;\;\;\;\;\;otherwise \end{array}\right..$$

In this model, each individual’s limited field of view in a three-dimensional space is defined as a cone-shaped region extending from their line of sight. The line of sight for an individual at each iteration is aligned with the direction of the velocity vector of the particle from the previous time step. Individuals interact only with their neighbours within this field. All individuals are assumed to share a uniform perspective, denoted by θ∈(0,π). When θ=π, the model reverts to a $360^{\circ }$ field of view and effectively functions as a basic SLH-SMF model with a prospective mechanism. In this modified model, $B_{iv}$ represents the topological neighbours within the field of view of individual i, whereas $S_{iv}$ denotes the neighbours on the convex hull of the cluster within this field of view.

To analyse the changes in coordination consistency within a collective system governed by the SLH-SMF model with limited vision, the field of view was varied from $15^{\circ }$ to $180^{\circ }$ in $15^{\circ }$ intervals. To ensure accurate and reliable results, the model parameters were optimized using the DE algorithm to determine the optimal values for different population sizes in the SLH-SMF model.

### 2.2. Multi-layer hierarchical strictly metric-free model

In natural biological populations, variations in leadership abilities among individuals often result in hierarchical structures. A notable example can be observed in penguin societies, where multiple penguins may assume leadership roles. Within such hierarchies, the most robust and intelligent penguin typically emerges as the primary leader, while others take on subordinate roles. The primary leader wields greater authority and guides the group through the directives.

This study incorporated a multi-layer leader–follower hierarchical mechanism into the SMF model. In this framework, each individual i on the convex hull is assigned a preferred direction weight $\omega_{i}$. Furthermore, variations in leaders’ decision-making proficiency introduce deviations in the preferred direction of the information received. Each individual i on the convex hull is characterized by a unique preference direction denoted as $g_{i}$.

In each independent repeated experiment, $\omega_{i}$ is randomly assigned as a floating-point number within the range [0,1), ensuring unique preference weights for all leader individuals on the convex hull. Similarly, the preference direction $g_{i}$ for each leader is distinct. To generate $g_{i}$, a three-dimensional unit vector is created, with each component being a random floating-point number in the range (−1,1). A random deviation is then introduced to this unit vector to generate a random preference direction, which is subsequently normalized. This ensures that each leader on the convex hull has a unique preference direction, reflecting the variability in leadership when processing external information. These configurations for $\omega_{i}$ and $g_{i}$ establish a multi-layer hierarchical mechanism model with varying leadership strengths.

The mathematical formulation of the multi-layer hierarchical SMF (MLH-SMF) model can be expressed through the following equations:


(2.14) $$r_{i}^{t+1}=r_{i}^{t}+v_{0}{\hat{v}}_{i}^{t},$$

(2.15) $$\begin{array}{lcr} & v_{i}^{t+1}=\left(1-\phi_{n}\right){\hat{u}}_{i}^{t}+\phi_{n}{\hat{\eta }}_{i}^{t} & \end{array}$$

and


(2.16) $$u_{i}^{t}=\left\{ \begin{array}{c} \left(1-\phi_{e}\right)\frac{{\left\langle {\hat{v}}_{j}^{t}\right\rangle }_{j\in B_{i}}\left(1-\omega_{i}\right)+\omega_{i}g_{i}}{\left|{\left\langle {\hat{v}}_{j}^{t}\right\rangle }_{j\in B_{i}}\left(1-\omega_{i}\right)+\omega_{i}g_{i}\right|}+f_{i}{\left\langle {\hat{r}}_{ij}^{t}\right\rangle }_{j\in S_{i}\;}\;\;\;\;\;\;\;\;\;\;\;\;\;\;i\in C\\ \frac{{\left\langle {\hat{v}}_{j}^{t}\right\rangle }_{j\in B_{i}}}{\left|{\left\langle {\hat{v}}_{j}^{t}\right\rangle }_{j\in B_{i}}\right|}\;\;\;\;\;\;\;\;\;\;\;\;\;\;\;\;\;\;\;\;\;\;\;\;\;\;\;\;\;\;\;\;\;\;\;\;\;\;\;\;\;\;\;\;\;\;\;\;\;\;\;\;\;\;\;\;\;\;\;\;\;\;\;\;\;\;\;\;\;otherwise \end{array}\right..$$

## 3. Results

### 3.1. Singular-layer hierarchical strictly metric-free model

#### 3.1.1. Models

**Figure 2.**
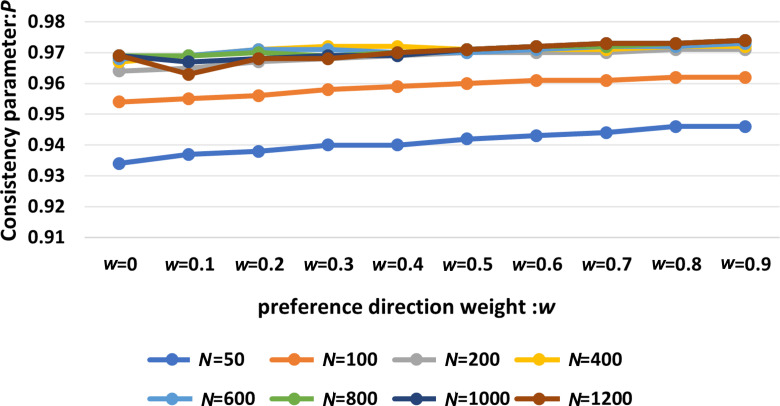
Consistency analysis of the SLH-SMF model governed by equations (2.1)–(2.3) with respect to the preference direction weight ω across different population sizes.

Figure 2 shows the simulation results of the SLH-SMF model governed by equations (2.1)–(2.3) to evaluate the impact of ω on the coordination consistency of the collective system under varying population sizes. Therein, the simulation parameters are listed in table 1. The horizontal axis of the figure shows 10 different values for ω. Here, ω=0 represents leaderless individuals, which corresponds to the basic SMF model. Values of ω ranging from 0.1 to 0.9 correspond to the SLH-SMF model with individual leaders. Compared with the basic SMF model (ω=0), the consistency and coordination of the swarming system in the SLH-SMF model improved continuously as ω increased, particularly in smaller populations where the improvement was more pronounced. For larger populations, the increase in consistency and cooperation after reaching stability was slower.

**Table 1.** Simulation parameters for changing the preference direction weight ω in the SLH-SMF model.

parameter	N	$\phi_{n}$	$\phi_{e}$	ω	$v_{0}$	iteration	test number
value	50, 100	0.2	0.5	0, 0.1, 0.2	1	1000	20
	200, 400	—	—	0.3, 0.4, 0.5	—	—	—
	600, 800	—	—	0.6, 0.7	—	—	—
	1000,1200	—	—	0.8, 0.9	—	—	—

#### 3.1.2. Parameter optimization

In the SLH-SMF model, this study aimed to optimize three critical control parameters: $\phi_{n}$, $\phi_{e}$ and ω. We set the dimension D=3 and used a parent population size of $N_{p}=30$ for the DE algorithm, resulting in 30 initial sets of $\phi_{n}$, $\phi_{e}$ and ω for optimization. The orderliness of the collective system after optimization was compared with that of the unoptimized system to assess the effectiveness of the DE algorithm.

An analysis of the experimental results for the optimized SLH-SMF model showed that the consistency parameter P exhibited minimal variation after approximately 10 rounds of optimization iterations. We selected the parameter value where the change in P was not greater than 0.0005 as the optimal solution for the model parameters. The results are presented in table 2.

**Table 2.** Optimal parameters $\phi_{e}$, $\phi_{n}$ and ω of the SLH-SMF model.

N	$\phi_{e}$	$\phi_{n}$	ω	P
50	0.11503	0.10608	0.77567	0.99529
100	0.35541	0.11057	0.78466	0.99435
200	0.10231	0.10015	0.60453	0.99542
400	0.16605	0.10178	0.41193	0.99467
600	0.13322	0.10235	0.34714	0.99492
800	0.16781	0.10027	0.28843	0.99507
1000	0.11820	0.10368	0.40064	0.99481
1200	0.10655	0.10198	0.25851	0.99495

**Figure 3.**
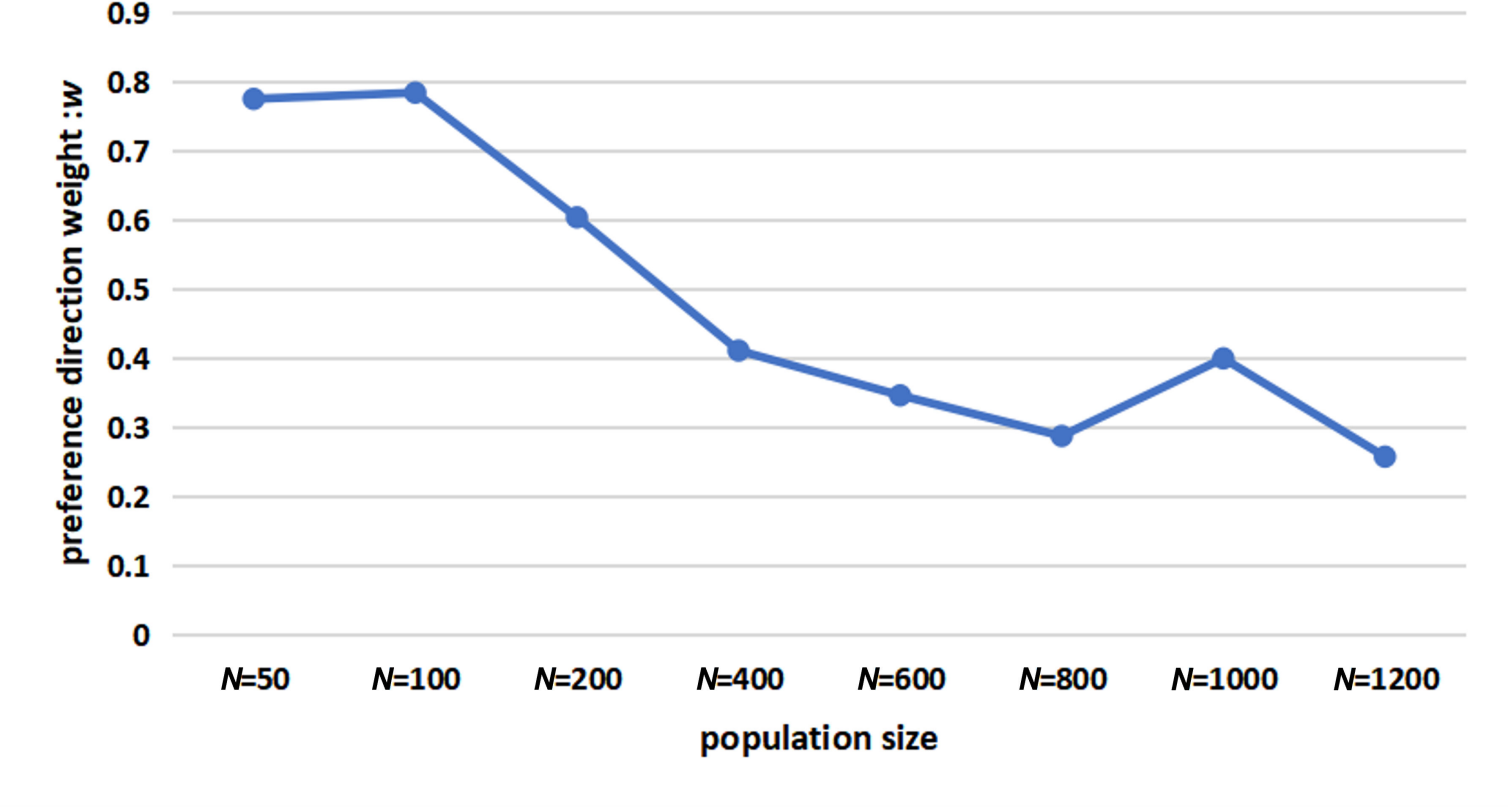
The optimal parameter ω of the SLH-SMF model under different population sizes (experimental control parameters: *F* = 0.8, CR = 0.55, Np= 30, rounds = 50).

Figure 3 illustrates the experimental findings and shows how the preference direction weight ω varies with different population sizes. In smaller populations, ω is higher, indicating greater reliance on leadership-driven decision-making.

**Figure 4.**
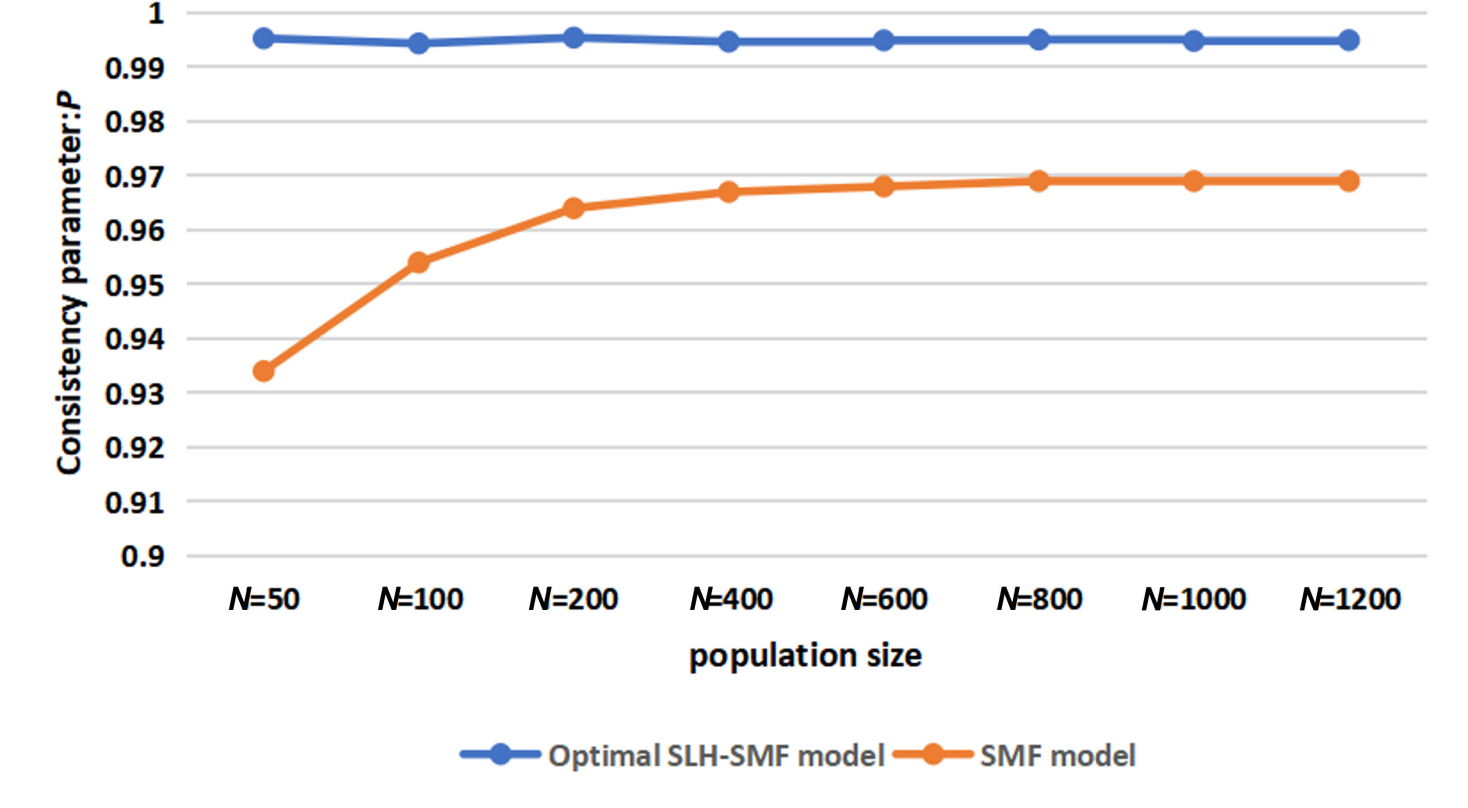
Consistency comparison between the optimal SLH-SMF model and the SMF model ($\phi_{n}=0.2$, $\phi_{e}=0.5$, ω=0) under different population sizes.

We simulated six sets of parameters for the SLH-SMF model, optimized using the DE algorithm, and compared them with the standard SMF model with parameters $\phi_{n}=0.2$, $\phi_{e}=0.5$ and ω=0. To validate the effectiveness of the parameter optimization algorithm, this comparison evaluated the degree of orderliness in the flocking system. Figure 4 shows that the SLH-SMF model with optimized control parameters achieves a higher degree of orderliness after stabilization than the SMF model with standard parameters.

#### 3.1.3. Model optimization

**Figure 5.**
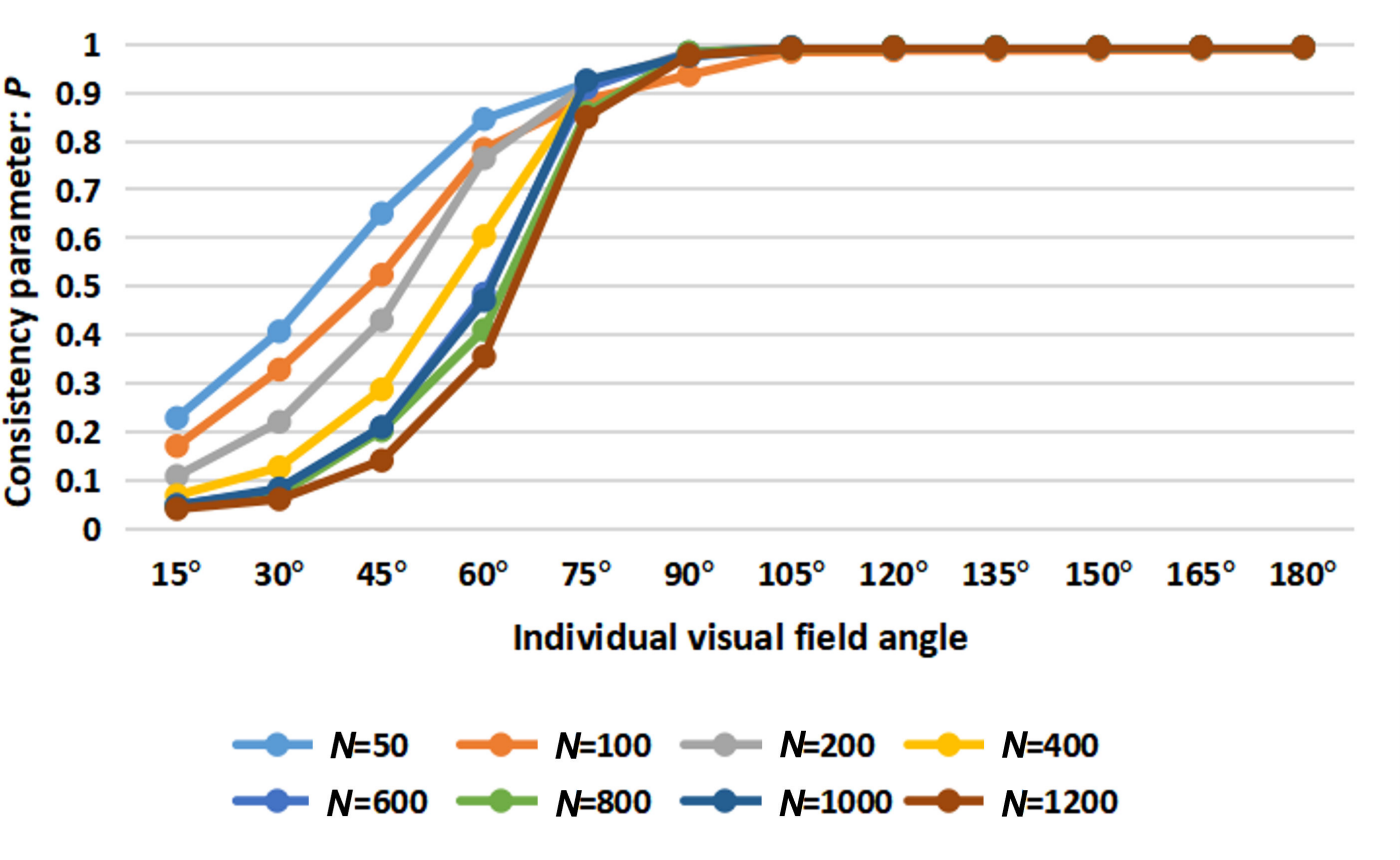
Consistency analysis of the SLH-SMF model versus varying visual angles under different population sizes.

Drawing inspiration from the limited vision of social animals such as starlings, we further optimize the SLH-SMF model by incorporating the perspective of visual angles. Consequently, a generalized SLH-SMF model with adjustable visual angles for each individual is proposed, as described in equations (2.11)–(2.13). A series of simulation experiments were conducted to investigate how the field of view influences the coordination and consistency of the system. The specific parameter settings are listed in table 3. The consistency parameter P is employed as the performance metric. As illustrated in figure 5, the simulation results demonstrate that, across different population sizes, coordination and consistency within the collective system improve with increasing field-of-view angles. Notably, when the visual angle exceeded $105^{\circ }$, the consensus level stabilizes and remains consistently high.

**Table 3.** Simulation parameters for changing visual angles in the SLH-SMF model.

parameter	N	$\phi_{n}$	$\phi_{e}$	ω	field angle	iteration	test number
value	50	0.10608	0.11503	0.77567	$15^{\circ }$ $30^{\circ }$	1000	20
	100	0.11057	0.35541	0.78466	$45^{\circ }$ $60^{\circ }$	—	—
	200	0.10015	0.10231	0.60453	$75^{\circ }$ $90^{\circ }$	—	—
	400	0.10178	0.16605	0.41193	$105^{\circ }$ $120^{\circ }$	—	—
	600	0.10235	0.13322	0.34714	$135^{\circ }$ $150^{\circ }$	—	—
	800	0.10027	0.16781	0.28843	$165^{\circ }$ $180^{\circ }$	—	—
	1000	0.10368	0.11820	0.40064	—	—	—
	1200	0.10198	0.10655	0.25851	—	—	—

### 3.2. Multi-layer hierarchical strictly metric-free model

To explore the impact of multi-layer hierarchical mechanisms on the collective dynamics of the system governed by equations (2.14)–(2.16), we first consider that each leader i on the convex hull has the same preference direction $g_{1}=g_{2}=...=g_{N}=g$ but a distinct, randomly generated preference direction weight $\omega_{i}$. Next, we analyse the scenario, where each leader i has the same preference direction weight $\omega_{1}=\omega_{2}=...=\omega_{N}=\omega$ but a different preference direction $g_{i}$. Finally, we discuss the case where each leader simultaneously has different preference directions $g_{i}$ and preference weights $\omega_{i}$.

#### 3.2.1. The influence of preference direction weight

The simulation experiments primarily aim to examine the impact of random preference direction weights $\omega_{i}$ on the coordination consistency of collective systems. In these experiments, the noise intensity $\phi_{n}$ was set to 0.2, the edge intensity $\phi_{e}$ to 0.5 and the preference direction weight $\omega_{i}$ varied between 0 and 1. Each simulation was run for 1000 steps, with the final 100 iterations used to calculate the consistency parameter P. To ensure the reliability of the results, 20 independent repetitions of each experiment were conducted.

**Figure 6.**
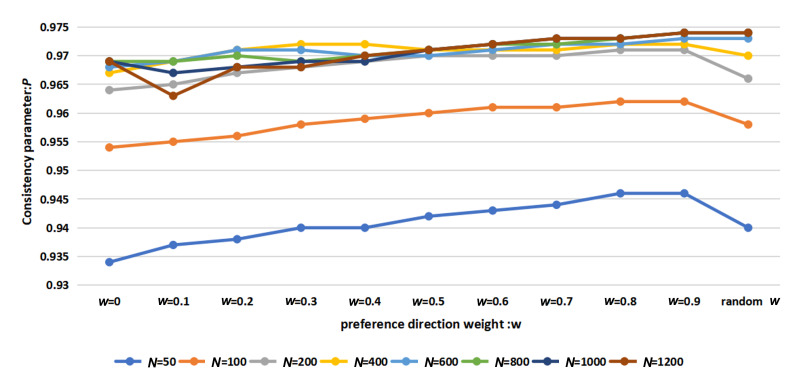
MLH-SMF models specifying preference direction weights and random values are compared for consistency in different population sizes (experimental control parameters: $\phi_{n}=0.2$, $\phi_{e}=0.5$, $\omega_{i}\in \left[0,1\right)$, iteration = 1000, test number = 20).

The experimental results are presented in figure 6, which compares the MLH-SMF model, where each leader has a distinct random preference direction weight, with the MLH-SMF model in which all leaders share a single given preference direction weight. Notably, when the given preference direction weight ω equals 0, it signifies that each leader does not rely on their own preferred direction when making decisions but is instead influenced by the average behaviour of their neighbours, which corresponds to the SMF model.

#### 3.2.2. The influence of preference direction

**Figure 7.**
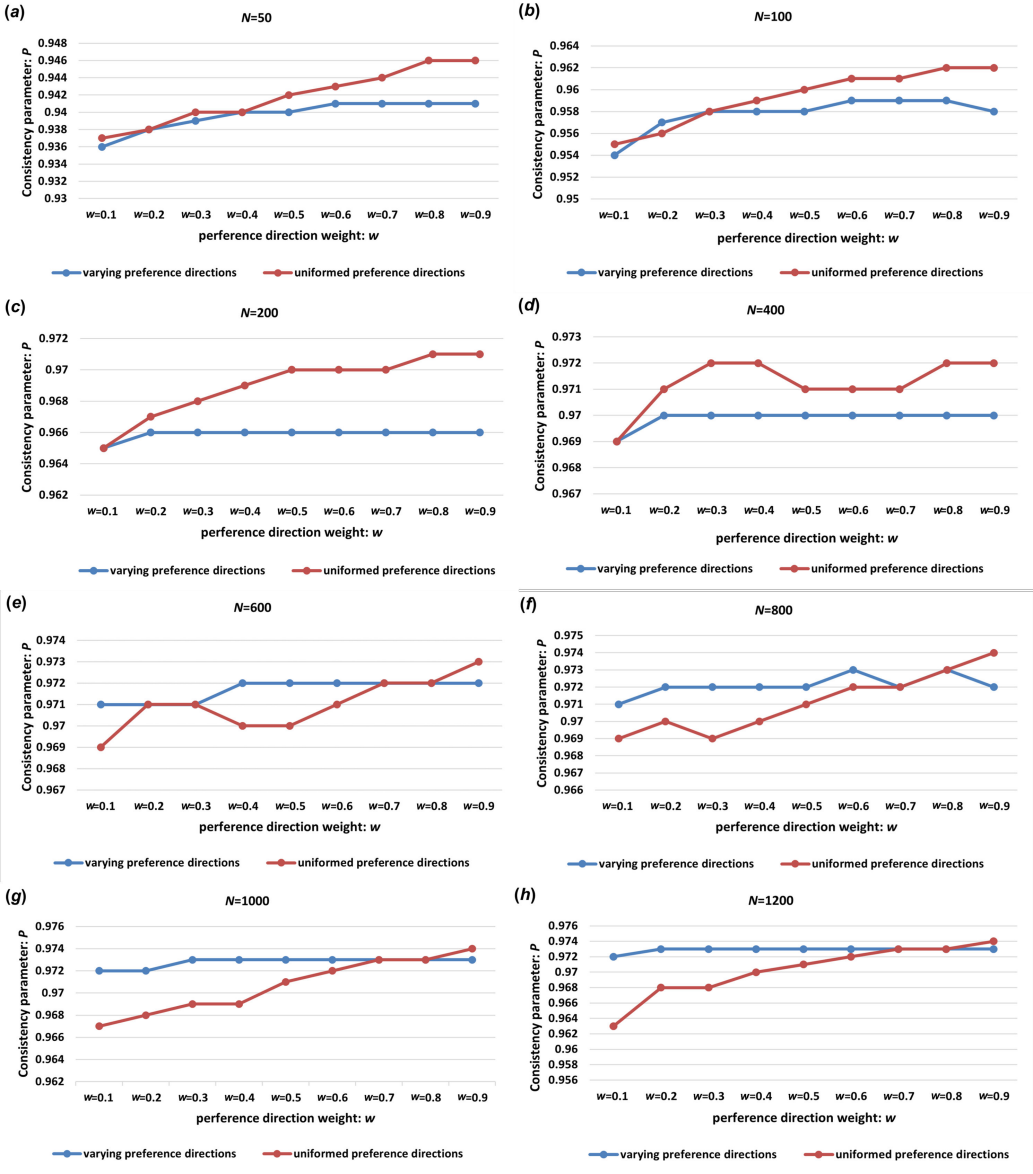
Consistency comparison of MLH-SMF models with different preference directions and uniform preference directions in different population sizes: (*a*) *N* = 50; (*b*) *N* = 100; (*c*) *N* = 200; (*d*) *N* = 400; (*e*) *N* = 600; (*f*) *N* = 800; (*g*) *N* = 1000; (*h*) *N* = 1200. (Experimental control parameters: $\phi_{n}=0.2$, $\phi_{e}=0.5$. The value of $\omega_{i}$ is 0.1, 0.2, 0.3, 0.4, 0.5, 0.6, 0.7, 0.8, 0.9, $g_{i}$ is the $15^{\circ }$ random deviation of g, iteration = 100 and test number = 20.)

In this experiment, the impact of the multi-layer hierarchical mechanism on the dynamics of the collective system is explored through the differing preference directions $g_{i}$ of the leaders i located on the convex hull. The primary objective is to investigate how random preference directions $g_{i}$ influence the coordination and consistency of the collective system across different population sizes. The experimental results are presented in figure 7.

During each experiment, all leaders on the convex hull are assigned the same base preference direction g. However, each leader exhibits a deviation in their acceptance of this direction. The random preference directions $g_{i}$ for each leader i are generated with a deviation value of $15^{\circ }$ from the given preference direction. To enable a comparative analysis of the collective models, the noise intensity $\phi_{n}$ was set to 0.2 and the edge intensity $\phi_{e}$ was set to 0.5. Figure 7 compares the MLH-SMF model, where each leader has a distinct random preference direction, with the MLH-SMF model in which all leaders share a uniform preference direction, across different population sizes.

#### 3.2.3. The influence of preference weight and preference direction

In the MLH-SMF model discussed in this section, a multi-layer hierarchical mechanism is introduced by allowing variability in both the preferred directions and preference weights of the leaders on the convex hull. Specifically, each leader has a random preferred direction denoted by $g_{i}$ and a corresponding preference weight $\omega_{i}$. This study investigates how the randomness in both the preferred direction $g_{i}$ and preference weight $\omega_{i}$ of leaders affects the coordination consistency of the collective system across different population sizes.

**Figure 8.**
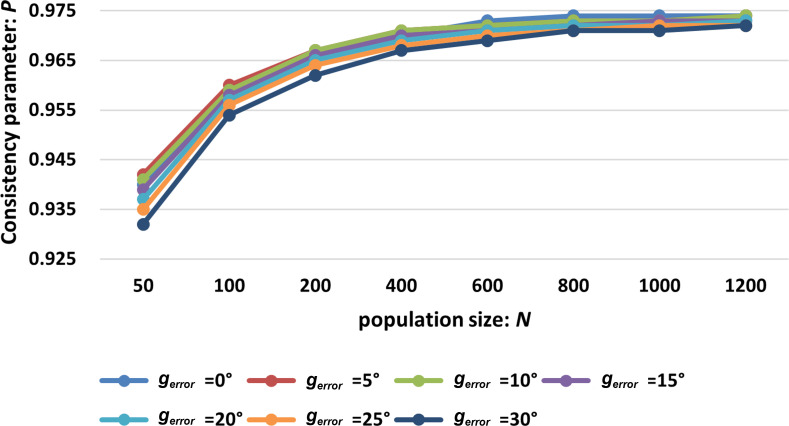
Consistency comparison between the MLH-SMF model (with random preference direction weights and random preference directions) and the SMF model under different population sizes. (MLH-SMF model control parameters: $\phi_{n}=0.2$, $\phi_{e}=0.5$, $\omega_{i}\in \left[0,1\right)$, $g_{error}=$ $5^{\circ }$,$10^{\circ }$,$15^{\circ }$,$20^{\circ }$,$25^{\circ }$,$30^{\circ }$, iteration = 1000, test number = 20; and SMF model control parameters: $\phi_{n}=0.2$, $\phi_{e}=0.5$, ω=0.)

The noise intensity $\phi_{n}$ was set to 0.2, and the edge intensity $\phi_{e}$ was set to 0.5. Variability in leaders’ decision-making abilities introduces deviations in their acceptance of the preferred direction, which, in turn, influences collective behaviour. In subsequent experiments, we explored the impact of this variability by considering error magnitudes of $5^{\circ }$, $10^{\circ }$, $15^{\circ }$, $20^{\circ }$, $25^{\circ }$ and $30^{\circ }$. These experiments aimed to understand how different levels of uncertainty in leaders’ preferred directions influence the system’s ability to maintain coordination consistency under various population sizes. The experimental results are presented in figure 8. To facilitate the presentation of the comparison results, the experimental data of the SMF model are also included in figure 8 as a reference.

## 4. Discussion

### 4.1. Singular-layer hierarchical strictly metric-free model

The results in figure 2 demonstrate that incorporating a leader–follower mechanism into the swarm model enhances the coordination and consistency of the system across all population sizes, supporting the presence of leaders in natural biological swarms.

For example, in smaller groups of lions, the leader’s guidance is crucial for activities such as migration and hunting. Conversely, in larger groups such as schools of sardines, individual interactions and collective decision-making play a more significant role. These phenomena are exactly consistent with the simulation results shown in figure 3.

Figure 4 confirms that the DE algorithm effectively identifies the optimal values for the three parameters across different population sizes, thereby enhancing the consistency and coordination of the flocking model. These results further validate the efficacy of the DE algorithm in optimizing flocking systems.

The simulation results shown in figure 5 indicate that an optimal perspective for individuals in a population does not correspond to a global view, regardless of the population size.

### 4.2. Multi-layer hierarchical strictly metric-free model

This study investigates the impact of the multi-layer hierarchical mechanism on the coordination consistency of collective behaviours. This study explores three dimensions: adjusting the preference direction weight for each individual, modifying individuals’ preference directions and simultaneously varying both the preference direction weight and preference direction. These adjustments were examined to understand their influence on the coordination behaviour and interaction structure of the collective system.

Figure 6 reveals that when the population size ranges from 50 to 400, the coordination consistency of the collective system with a leader individual assigned a random preference direction weight on the convex hull is less effective than when a larger, consistent value is used. However, the introduction of a multi-layer hierarchical mechanism with random preference direction weights still improves the coordination consistency of the collective system compared with the basic SMF model. Notably, when the population size exceeded 600, the leader individual with a random preference direction weight on the convex hull achieved a better-coordinated state within the collective system. There exists an optimal trade-off between group size and preference direction weight. That is to say, to achieve the same level of coordination and consistency within the collective system, smaller populations require leaders to exhibit a stronger collective inclination, which corresponds to smaller reference direction weights. Conversely, larger populations benefit from leaders having stronger individuality and greater self-reliance, which corresponds to larger reference direction weight.

Figure 7 reveals that for smaller populations ranging from 50 to 400, the coordination consistency of the collective system is less effective when the leader individual on the convex hull has a random preference direction compared with a uniform preference direction. In such cases, it is advantageous for the leader to maintain consistent preference directions. Conversely, in larger populations of 600 to 1200, a leader with a random preference direction on the convex hull can achieve a higher level of coordination consistency within the collective system.

Figure 8 demonstrates that as the population size increases, the negative impact of cognitive offsets in the preferred direction of leader individuals on the cooperative consistency of the swarm system diminishes. When the maximum population size N=1200 and the cognitive offset was within the range of $0^{\circ }$ to $10^{\circ }$, the swarm model achieved the highest degree of cooperative consistency. This finding aligns with the analysis shown in figure 7, where it was observed that in larger populations, random preference directions contribute to a higher degree of cooperative consistency once the swarm system stabilizes. Furthermore, the results indicate that compared with the basic SMF swarm model, the introduction of the multi-layer hierarchical mechanism with random preference direction weights and random preference directions (with an offset of $0^{\circ }$ to $25^{\circ }$) can enhance the coordination consistency of the swarm system.

## 5. Conclusion

In this study, we have explored coordination efficacy within diverse hierarchical layers under the SMF paradigm, building upon the foundational SMF model by incorporating leader–follower dynamics. By applying the DE algorithm, we optimized the SLH-SMF frameworks across varying population scales. Our findings underscore the critical importance of leadership directionality weight in achieving a consensus within the system. Specifically, increased weights enhance coordination in smaller groups, whereas reduced weights are advantageous for larger populations. Furthermore, this research introduces a limited field of view into the SLH-SMF model, revealing that high levels of coordination and consistency can be maintained across all population sizes, provided that the visual angle is not less than $105^{\circ }$. This challenges the prevailing assumption that a $360^{\circ }$ field of view is necessary for effective coordination. Additionally, we examined the impact of multi-layer hierarchical structures on coordination fidelity, discovering complex variations influenced by population size and leadership preference characteristics. The results of this study strongly support the notion that biological diversity is a significant factor in natural ecosystems.

## Data Availability

Relevant codes for this research work are available online [39].
